# An Aided Navigation Method Based on Strapdown Gravity Gradiometer

**DOI:** 10.3390/s21030829

**Published:** 2021-01-27

**Authors:** Duanyang Gao, Baiqing Hu, Lubin Chang, Fangjun Qin, Xu Lyu

**Affiliations:** Department of Navigation Engineering, Naval University of Engineering, Wuhan 430000, China; gdyhgn@163.com (D.G.); changlubin@163.com (L.C.); haig2005@126.com (F.Q.); lvclay@163.com (X.L.)

**Keywords:** strapdown gravity gradiometer, strapdown inertial navigation system, quaternion, unscented Kalman filter, gravity gradient aided navigation

## Abstract

The gravity gradient is the second derivative of gravity potential. A gravity gradiometer can measure the small change of gravity at two points, which contains more abundant navigation and positioning information than gravity. In order to solve the problem of passive autonomous, long-voyage, and high-precision navigation and positioning of submarines, an aided navigation method based on strapdown gravity gradiometer is proposed. The unscented Kalman filter framework is used to realize the fusion of inertial navigation and gravity gradient information. The performance of aided navigation is analyzed and evaluated from six aspects: long voyage, measurement update period, measurement noise, database noise, initial error, and inertial navigation system device level. When the parameters are set according to the benchmark parameters and after about 10 h of simulation, the results show that the attitude error, velocity error, and position error of the gravity gradiometer aided navigation system are less than 1 arcmin, 0.1 m/s, and 33 m, respectively.

## 1. Introduction

In view of the cost of gravity gradiometers and the existence of outdoor global satellite navigation systems, more attention is paid to the special application scenarios of gravity matching under the condition of satellite rejection, especially in the military application field. Submarines adopt an integrated navigation scheme based on inertial navigation and assisted by other navigation means. When global navigation satellite systems, Loran-C, and celestial navigation are used to correct an inertial navigation system (INS), the submarine needs to be in periscope navigation state, and the concealment of the submarine cannot be guaranteed. With the aid of sonar and Doppler velocity log, the velocity and altitude information of the INS can be corrected, but the acoustic signal is broadcasted, so it is easy to expose the submarine’s position to the enemy. Through building a base station, submarine positioning in a certain sea area can be realized, but the process of base station construction and maintenance is complicated, and it does not have the ability of positioning in the whole sea area. An underwater emergency positioning buoy is also a kind of submarine aided navigation means, but this method has high economic cost and is usually only used in emergency situations. So, it has been a difficult problem in the field of navigation on how to realize submarine passive autonomous, long-voyage and high-precision navigation and positioning [1,2,3,4].

The aided navigation method based on the geophysical field provides a new idea and means to solve this problem [5,6,7]. The marine gravity field and geomagnetic field are two kinds of geophysical fields that we are mainly concerned about. As the geomagnetic field belongs to weak field signals, it is easy to be interfered with by other factors, especially by the submarine shell. The geomagnetic measurement is usually carried out by a dragging mode, which provides a poor positioning accuracy of several kilometers [8,9,10] of the submarine navigation system based on geomagnetic field signals. In addition, the geomagnetic real-time measurement is more troublesome. Compared with the geomagnetic field, the gravity field signal has stronger anti-interference and stability, and is more suitable for underwater, providing aided navigation information for inertial navigation systems [11,12,13,14]. The gravity gradient is the second derivative of the gravity potential. Relative to gravity, a gravity gradiometer can measure subtle changes of gravity signals in different geographical locations [15,16]. Therefore, gravity gradient signals contain more abundant navigation information.

The gravity gradient database, gravity gradiometer, and gravity gradient matching algorithm are the three key parts affecting the gravity information aided navigation system, and most of the research work is based on the above three factors. Over the past few decades, gravity models of terrestrial planets, especially the Earth, have been improved dramatically. Derived from the combination of satellite geodetic data with high-resolution gravitational information collected from surface gravimetry, the Earth Gravitational Model 2008 (EGM2008) is complete to degree 2190 and order 2159, which is a representative of the high-resolution models. The high order gravity field model based on EGM2008 is an effective method to establish the global gravity information database. In the future, the gravity gradiometer with atomic interference technology can theoretically realize higher precision gravity gradient measurement [17]. In previous studies, the extended Kalman filter framework was used to realize gravity gradient aided navigation (GGAN) [18,19,20]. In this framework, the mathematical model needs to be linearized. Some particle filter algorithms have also been considered in the field of gravity gradient matching algorithm [21,22]. In addition, most of the previous literatures studied the mathematical model and performance analysis based on the platform gravity gradiometer, and some factors affecting performance were considered [23,24]. However, it is necessary to investigate the performance of applying the strapdown gravity gradiometer and systematically evaluate the performance of GGAN under the influence of various factors, and there is still a need to verify the effects of some other factors, such as long voyage and inertial navigation system device level.

In this study, we focus on an unscented quaternion estimator, which has a better estimation accuracy, confirmed by previous studies [25], and establish a framework of unscented Kalman filter (UKF) based on strapdown gravity gradiometer to realize GGAN. Simulation experiments were conducted for systematically evaluating the performance of GGAN under the influence of various factors, such as long voyage, measurement update period, measurement noise, database noise, initial errors (attitude, velocity, and position), and inertial navigation system device level. As a result, we drew some numerical conclusions, which can provide some suggestions for obtaining navigation results with some target accuracies in GGAN on the basis of the simulation results.

The organization of this paper is as follows. In the review of existing theories, the basic strapdown inertial navigation system (SINS) equations and the principle of gravity gradiometer are introduced. In the gravity gradient matching algorithm section, UKF is studied in detail. Finally, simulation testing is conducted and is summarized in the experimental section. The results are provided and the performance of the six factors are evaluated. A few meaningful conclusions are outlined in the summary.

## 2. Basic Equations of Strapdown Inertial Navigation System

The basic equations of strapdown inertial navigation system (SINS) include attitude differential equation, velocity differential equation, position differential equation, gyroscope error differential equation, and accelerometer error differential equation. Compared with the platform inertial navigation system, SINS uses the “mathematical platform” simulation navigation coordinate system. In the basic equation of SINS, “mathematical platform” is determined by the attitude transfer matrix $C_{b}^{n}$ and the attitude differential equation. Different attitude expression methods correspond to different attitude differential equations. Since quaternion attitude expression is continuous and has no singularity problem, this paper adopts quaternion representation, and its differential equation is generally expressed as follows:

(1) $$\dot{q}=\frac{1}{2}\Xi (q)\omega_{nb}^{b}=\frac{1}{2}\Omega (\omega_{nb}^{b})q,$$

where $q=\left[q_{0};\rho \right]$ is the quaternion, where $\rho =\left[q_{1};q_{2};q_{3}\right]$ is the vector part, and $q_{0}$ is the scalar part, and

(2) $$\Omega (\omega_{nb}^{b})=\left[\begin{array}{cc} -[\omega_{nb}^{b}\times ] & \omega_{nb}^{b}\\ -{\left(\omega_{nb}^{b}\right)}^{T} & 0 \end{array}\right]$$

Other parts of the basic SINS equations are

(3) $$\dot{L}=\frac{v_{N}}{R_{M}+h},$$

(4) $$\dot{\lambda }=\frac{v_{E}}{(R_{N}+h)\cos L},$$

(5) $$\dot{h}=v_{U},$$

(6) $${\dot{v}}_{E}=\left[\frac{v_{E}}{(R_{N}+h)\cos\lambda }+2\omega_{ie}^{e}\right]v_{N}\sin\lambda -\frac{v_{E}v_{U}}{R_{N}+h}-2\omega_{ie}^{e}v_{U}\cos\lambda +f_{E},$$

(7) $${\dot{v}}_{N}=-\left[\frac{v_{E}}{(R_{N}+h)\cos\lambda }+2\omega_{ie}^{e}\right]v_{E}\sin\lambda -\frac{v_{E}v_{U}}{R_{M}+h}+f_{N},$$

(8) $${\dot{v}}_{U}=\frac{v_{E}^{2}}{R_{N}+h}+\frac{v_{N}^{2}}{R_{M}+h}+2\omega_{ie}^{e}v_{E}\cos\lambda -g-f_{U}$$

Equations (3)–(5) are the position differential equation. $p=\left[L;\lambda ;h\right]$ is the position information, where L is the latitude, λ is the longitude, and h is the height; Equations (6)–(8) are the velocity differential equations. $v=\left[v_{E}^{};v_{N}^{};v_{U}^{}\right]$ represents velocity information, where $v_{E}^{}$ represents eastward velocity, $v_{N}^{}$ represents northward velocity, and $v_{U}^{}$ represents vertical velocity; g is the acceleration of gravity; $f=[f_{E};f_{N};f_{U}]$ is the specific force in the navigation coordinate system. The output of the gyroscope is $\omega_{ib}^{b}$; $R_{M}$ and $R_{N}$ represent the meridian and prime vertical radius of the Earth, as shown in the following formula:

(9) $$R_{M}={R_{e}(1-e^{2})/\left(1-e^{2}\sin^{2}\lambda \right)}^{1.5},$$

(10) $$R_{N}={R_{e}/\left(1-e^{2}\sin^{2}\lambda \right)}^{0.5},$$

where $R_{e}$ = 6378137m, e = 0.0818.

The differential equation of gyro constant drift is as follows:

(11) $${\tilde{\omega }}_{ib}^{b}=\omega_{ib}^{b}+\varepsilon +\eta_{gv},$$

(12) $$\dot{\varepsilon }=\eta_{gu},$$

where ${\tilde{\omega }}_{ib}^{b}$ is the actual gyro output with gyro drift ε; $\eta_{gv}$ and $\eta_{gu}$ are zero mean Gaussian white noise with covariance $\sigma_{gv}^{2}$ and $\sigma_{gu}^{2}$, respectively.

The output equation of the accelerometer reads as follows:

(13) $${\tilde{f}}^{b}=f^{b}+\nabla +\eta_{av},$$

(14) $$\dot{\nabla }=\eta_{au},$$

where ${\tilde{f}}^{b}$ is the actual accelerometer output with accelerometer bias ∇, $\eta_{av}$ and $\eta_{au}$ are zero mean Gaussian white noise with covariance $\sigma_{av}^{2}$ and $\sigma_{au}^{2}$, respectively.

Equations (3)–(14) together constitute the basic equation of SINS. By analyzing the characteristics of the equation, we can find that the basic equation of SINS is a nonlinear equation, if the filtering framework is used for information fusion, the integrated navigation needs to adopt nonlinear filtering processing.

## 3. Measurement Principle of Gravity Gradiometer

The gravity gradiometer is basically a differential accelerometer. The measurement principle is described in detail below.

Suppose that there exists an arbitrary coordinate system a, which rotates around the inertial coordinate system with angular velocity $\omega_{ia}^{a}$. If the position vector of a certain point is $r^{a}$ and the position vector of the point in the inertial coordinate system is $r^{i}$; $r^{i}$ can be calculated from

(15) $$r^{i}=C_{a}^{i}r^{a},$$

where $C_{a}^{i}$ is the transformation matrix between coordinate system a and inertial coordinate system i. It can be obtained by time derivation of Equation (15)

(16) $${\dot{r}}^{i}=C_{a}^{i}\Omega_{ia}^{a}r^{a}+C_{a}^{i}{\dot{r}}^{a},$$

if $\omega_{ia}^{a}={\left[\begin{array}{ccc} \omega_{1} & \omega_{2} & \omega_{3} \end{array}\right]}^{T}$, the antisymmetric matrix $\Omega_{ia}^{a}$ can be expressed as

(17) $$\Omega_{ia}^{a}=\left[\begin{array}{ccc} 0 & -\omega_{3} & \omega_{2}\\ \omega_{3} & 0 & -\omega_{1}\\ -\omega_{2} & \omega_{1} & 0 \end{array}\right]$$

The time derivative of Equation (16) leads to

(18) $${\ddot{r}}^{i}=C_{a}^{i}{\ddot{r}}^{a}+2C_{a}^{i}\Omega_{ia}^{a}{\dot{r}}^{a}+C_{a}^{i}(\Omega_{ia}^{a}\Omega_{ia}^{a}+{\dot{\Omega }}_{ia}^{a})r^{a}$$

In inertial space the following relation holds

(19) $${\ddot{r}}^{i}=a^{i}+g^{i},$$

where $a^{i}$ is the specific force output of the accelerometer and $g^{i}$ is the gravity of the Earth. Combining Equations (18) and (19) yields

(20) $$a^{a}={\ddot{r}}^{a}+2\Omega_{ia}^{a}{\dot{r}}^{a}+(\Omega_{ia}^{a}\Omega_{ia}^{a}+{\dot{\Omega }}_{ia}^{a})r^{a}-g^{a}$$

The two accelerometers are fixed at point A and point B in the coordinate system a, respectively, and the baseline of the two accelerometers is set as $\rho^{a}=r_{2}^{a}-r_{1}^{a}$. According to Equation (20), we can get

(21) $$a_{2}^{a}-a_{1}^{a}={\ddot{\rho }}^{a}+2\Omega_{ia}^{a}{\dot{\rho }}^{a}+(\Omega_{ia}^{a}\Omega_{ia}^{a}+{\dot{\Omega }}_{ia}^{a})\rho^{a}-(g_{2}^{a}-g_{1}^{a})$$

Since the accelerometer is attached to coordinate system a, then ${\dot{\rho }}^{a}={\ddot{\rho }}^{a}=0$. Both quantities can be eliminated from Equation (21)

(22) $$a_{2}^{a}-a_{1}^{a}=(\Omega_{ia}^{a}\Omega_{ia}^{a}+{\dot{\Omega }}_{ia}^{a})\rho^{a}-(g_{2}^{a}-g_{1}^{a})$$

The longer the baseline is, the more obvious the change of gravity signal is. Limited to the size of the gravity gradiometer, the length of baseline usually does not exceed 1 m. Therefore, it can be considered that the gravity between the two accelerometers varies linearly. The following relation can be obtained

(23) $$g_{2}^{a}-g_{1}^{a}=V^{a}(r_{2}^{a}-r_{1}^{a})=V^{a}\rho^{a},$$

where $V^{a}$ is the total tensor matrix of the gravity gradient in coordinate system a. By substituting Equation (23) into Equation (22), we obtain

(24) $$\begin{array}{ll} a_{2}^{a}-a_{1}^{a} & =(-V^{a}+\Omega_{ia}^{a}\Omega_{ia}^{a}+{\dot{\Omega }}_{ia}^{a})\rho^{a}\\ & =L^{a}{}^{\prime }\rho^{a} \end{array}$$

and

(25) $$L^{a}{}^{\prime }=(a_{2}^{a}-a_{1}^{a})/\rho^{a}=(a_{2}^{a}-a_{1}^{a})/(r_{2}^{a}-r_{1}^{a})$$

Equation (25) is the basic equation of gravity gradient based on the measurement principle of differential accelerometer. It can be seen from Equations (24) and (25) that the gravity gradient tensor cannot be directly measured, and the gradients are coupled with angular velocity $\Omega_{ia}^{a}$ and angular acceleration ${\dot{\Omega }}_{ia}^{a}$. In order to get the current gravity gradient, the angular velocity and angular acceleration should be eliminated. The angular acceleration component can be eliminated by summation of Equation (25) and its transposition

(26) $$\frac{1}{2}(L^{a}{}^{\prime }+{\left(L^{a}{}^{\prime }\right)}^{T})=-V^{a}+\Omega_{ia}^{a}\Omega_{ia}^{a}$$

Let

(27) $$L^{a}=-V^{a}+\Omega_{ia}^{a}\Omega_{ia}^{a}$$

By substituting each component into Equation (24), we end up with

(28) $$L^{a}=\left[\begin{array}{ccc} -(V_{xx}+\omega_{y}^{2}+\omega_{z}^{2}) & -(V_{xy}-\omega_{x}\omega_{y}) & -(V_{xz}-\omega_{x}\omega_{z})\\ -(V_{xy}-\omega_{x}\omega_{y}) & -(V_{yy}+\omega_{x}^{2}+\omega_{z}^{2}) & -(V_{yz}-\omega_{y}\omega_{z})\\ -(V_{xz}-\omega_{x}\omega_{z}) & -(V_{yz}-\omega_{y}\omega_{z}) & -(V_{zz}+\omega_{x}^{2}+\omega_{y}^{2}) \end{array}\right]$$

The six independent components in expression (28) are as follows:

(29) $$L^{a}=\left[\begin{array}{c} L_{11}^{a}\\ L_{22}^{a}\\ L_{33}^{a}\\ L_{12}^{a}\\ L_{13}^{a}\\ L_{23}^{a} \end{array}\right]=\left[\begin{array}{c} -(V_{xx}+\omega_{y}^{2}+\omega_{z}^{2})\\ -(V_{yy}+\omega_{x}^{2}+\omega_{z}^{2})\\ -(V_{zz}+\omega_{x}^{2}+\omega_{y}^{2})\\ -(V_{xy}-\omega_{x}\omega_{y})\\ -(V_{xz}-\omega_{x}\omega_{z})\\ -(V_{yz}-\omega_{y}\omega_{z}) \end{array}\right]$$

Compared with Equation (25), Equation (27) does not need to estimate ${\dot{\Omega }}_{ia}^{a}$, which is conducive to the extraction of gravity gradient tensor. So, Equation (27) is taken as the main measurement result of gravity gradiometer.

When the gradiometer is fixed to the carrier (strapdown mounting), the body coordinate system and acceleration coordinate system are the same coordinate system, so Equation (27) can be rewritten into

(30) $$L^{b}=C_{n}^{b}V^{n}{\left(C_{n}^{b}\right)}^{T}-\Omega_{ib}^{b}\Omega_{ib}^{b},$$

where $C_{n}^{b}$ is the transformation matrix between the navigation coordinate system and the body coordinate system, it can be obtained by attitude calculation, and $\Omega_{ib}^{b}$ can be retrieved from the gyro measurements. In this paper, Equation (30) is used as the measurement equation for simulation, and the calculation formula of $V^{n}$ is shown in the appendix.

## 4. Gravity Gradiometer Aided Navigation Method (GGAN Method)

The UKF algorithm abandons the linearization process of EKF algorithm, and adopts unscented transform (UT) to avoid the error caused by linearization, reduce the complexity of the algorithm, and overcome the defects of low accuracy and poor stability of the EKF algorithm. So, it is widely used in integrated navigation [20]. In this paper, the strapdown gravity gradiometer is used to measure the total tensor gravity gradient data. Therefore, the measurement vector is

(31) $$Z_{k}={\left[\begin{array}{ccc} L_{11}^{b} & L_{22}^{b} & \begin{array}{ccc} \begin{array}{cc} L_{33}^{b} & L_{12}^{b} \end{array} & L_{13}^{b} & L_{23}^{b} \end{array} \end{array}\right]}^{T},$$

where the superscript b represents the projection of the gravity gradient in the body coordinate system.

The state variables are the attitude φ, velocity v, and position p of the target and the drift vector b of the 6D gyroscope and accelerometer. Therefore, the state vector is

(32) $$X_{k}={\left[\begin{array}{cccc} \varphi & v & p & b \end{array}\right]}^{T}$$

At the kth moment, the system noise is Gaussian white noise $w_{k}\sim N(0Q_{k})$, and the measurement noise is Gaussian white noise $v_{k}\sim N(0R_{k})$. The nonlinear system of gravity gradient aided positioning can be expressed by the following formula:

(33) $$\left\{\begin{array}{l} X_{k}=F(X_{k-1})+w_{k-1}\\ Z_{k}=H(X_{k})+v_{k} \end{array}\right.$$

Firstly, the root mean square $S_{k-1}^{+}$ of the error covariance matrix needs to be solved in the UKF propagation process. This step can be conducted via the Cholesky decomposition

(34) $$P_{k-1}^{+}=S_{k-1}^{+}S_{k-1}^{+}{}^{T}$$

Calculate the sigma point from the following formula:

(35) $$X_{k-1}^{+(i)}=\left\{\begin{array}{l} {\hat{X}}_{k-1}^{+}+\sqrt{n}S_{k-1:,i}^{+},i\leq n\\ {\hat{X}}_{k-1}^{+}-\sqrt{n}S_{k-1:,(i-n)}^{+},i>n \end{array}\right.,$$

where the subscript “:,i” denotes the column i of the matrix. Each sigma point can be propagated through the system model:

(36) $$X_{k}^{-(i)}=F(k,X_{k-1}^{+(i)})$$

After propagation, the state estimation and its error covariance are as follows:

(37) $${\hat{X}}_{k}^{-}=\frac{1}{2n}\sum_{i=1}^{2n}X_{k}^{-(i)},$$

(38) $$P_{k}^{-}=\frac{1}{2n}\sum_{i=1}^{2n}(X_{k}^{-(i)}-{\hat{X}}_{k}^{-}){\left(X_{k}^{-(i)}-{\hat{X}}_{k}^{-}\right)}^{T}+Q_{k-1}$$

The observation update process of UKF generates new sigma points by the following formula:

(39) $$\begin{array}{ll} & P_{k}^{-}=S_{k}^{-}S_{k}^{-}{}^{T}\\ X_{k}^{-(i)}= & \left\{\begin{array}{l} {\hat{X}}_{k}^{-}+\sqrt{n}S_{k,:,i}^{-},i\leq n\\ {\hat{X}}_{k}^{-}-\sqrt{n}S_{k,:,(i-n)}^{-},i>n \end{array}\right. \end{array}$$

The sigma point and mean observed innovation can be solved by the following formula:

(40) $$\left\{\begin{array}{l} \delta Z_{k}^{-(i)}=Z_{k}-H(k,{\hat{X}}_{k}^{-(i)})\\ \delta Z_{k}^{-}=\frac{1}{2n}\sum_{i=1}^{2n}\delta Z_{k}^{-(i)} \end{array}\right.,$$

and the covariance of the observed innovation is

(41) $$C_{\delta Z,k}^{-}=\frac{1}{2n}\sum_{i=1}^{2n}(\delta Z_{k}^{-(i)}-\delta Z_{k}^{-}){\left(\delta Z_{k}^{-(i)}-\delta Z_{k}^{-}\right)}^{T}+R_{k}$$

Finally, the UKF Kalman gain, state vector update, and error covariance update can be calculated as follows:

(42) $$K_{k}=[\frac{1}{2n}\sum_{i=1}^{2n}(X_{k}^{-(i)}-{\hat{X}}_{k}^{-}){\left(\delta Z_{k}^{-(i)}-\delta Z_{k}^{-}\right)}^{T}]{\left(C_{\delta Z,k}^{-}\right)}^{-1},$$

(43) $${\hat{X}}_{k}^{+}={\hat{X}}_{k}^{-}+K_{k}\delta Z_{k}^{-},$$

(44) $$P_{k}^{+}=P_{k}^{-}-K_{k}C_{\delta Z,k}^{-}K_{k}^{T}$$

When the system noise and measurement noise are Gaussian white noise, GGAN can be realized by the standard UKF localization algorithm mentioned above.

## 5. Performance Analysis

Since the 21st century, with the continuous maturity and development of satellite altimetry, airborne gravimetry, and other gravity measurement technologies, the resolution and accuracy of the spherical harmonic function model of the Earth’s gravity field have been continuously improved. The most representative one is the EGM2008 ultra-high order spherical gravity field spherical harmonic model, issued by the National Geospatial-Intelligence Agency [26]. EGM2008 is a fusion of the ITG-GRACE03S model and global 5′ × 5′ gravity anomaly grid data (including land gravity survey, ocean satellite altimetry, and airborne gravity survey). In this paper, the EGM2008 model is used to simulate the gravity gradient, and the 360 order is intercepted to calculate the gravity gradient. The specific calculation formula is shown in Appendix A.

In order to evaluate the performance of gravity gradiometer aided navigation, this paper comprehensively considers the influence of long voyage, measurement update period, measurement noise, database noise, initial error, inertial device level, and other factors, in which the inertial device level mainly refers to gyro bias. In this paper, the database was calculated by the spherical harmonic model, and the calculated results were taken as the true value. To mimic a real situation, we introduced noise on the basis of the calculated values of the spherical harmonic model to simulate the actual database. The benchmark parameter settings are shown in Table 1 below.

### 5.1. Performance Analysis under Long Voyage Condition

**Figure 1:**
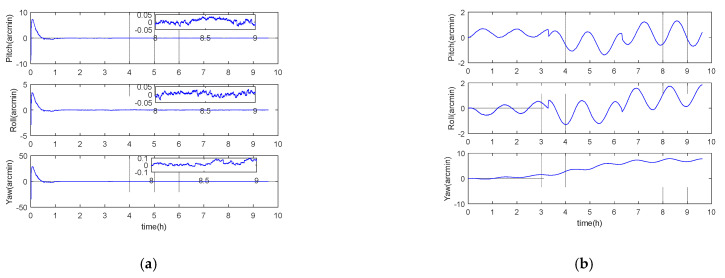
(**a**) Gravity gradient aided navigation (GGAN) attitude error; (**b**) pure inertial calculation attitude error.

**Figure 2:**
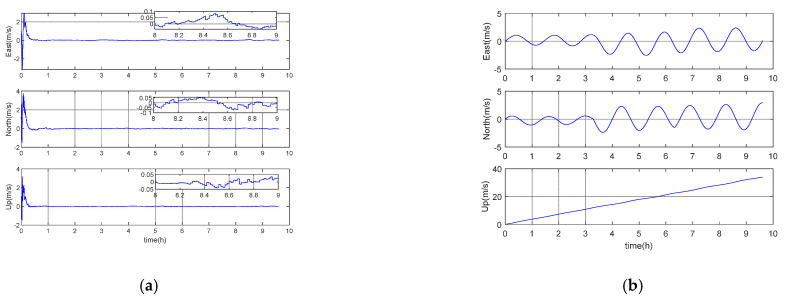
(**a**) GGAN speed error; (**b**) pure inertial calculation speed error.

**Figure 3:**
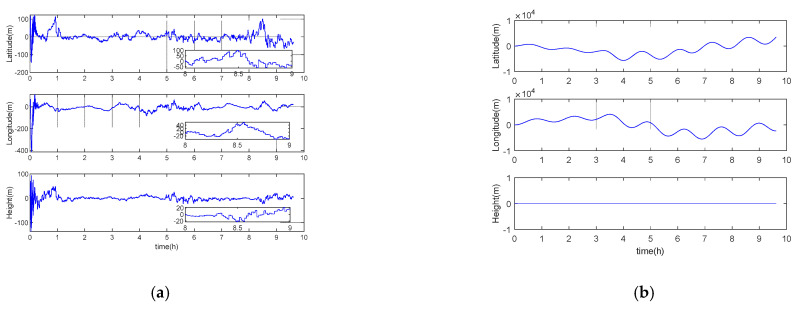
(**a**) GGAN position error; (**b**) pure inertial calculation position error.

In order to intuitively reflect the long voyage characteristics of inertial/gravity gradient integrated navigation, the simulation experiment designs acceleration, uniform speed, deceleration, steering, and other motion forms, and the navigation area is arbitrarily selected. The simulation step size is 0.01 s, and the total simulation time is about 6.5 h. Other parameters are set according to the benchmark parameters in Table 1. It should be noted that in the pure inertial calculation process, the altitude information is always set to zero, and the experimental results are shown in Figure 1, Figure 2 and Figure 3 and Table 2 below.

It can be seen from Figure 1, Figure 2 and Figure 3 that the error of inertial navigation system is effectively suppressed by GGAN. Both attitude and speed and position accuracy have been greatly improved, and the positioning error presents zero mean distribution. According to Table 2, after about 10 h of pure inertial calculation, the position errors in latitude and longitude directions are up to 2640 m and 2258 m, respectively. However, the position performance of GGAN method is two orders of magnitude better than that of pure inertial calculation method, and the attitude error, velocity error, and position error in all directions of GGAN system are less than 1 arcmin, 0.1 m/s, and 33 m, respectively.

### 5.2. Performance Analysis of Measurement Update Period

**Figure 4:**
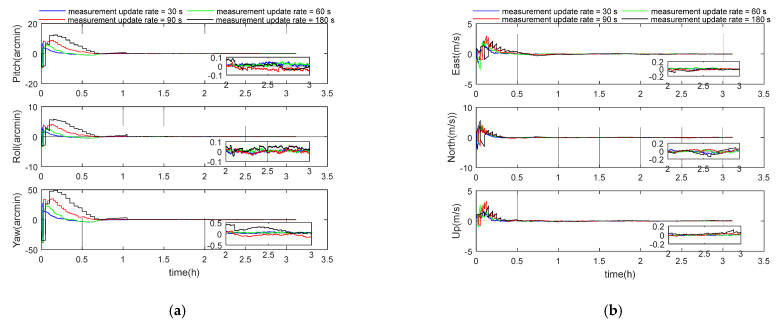

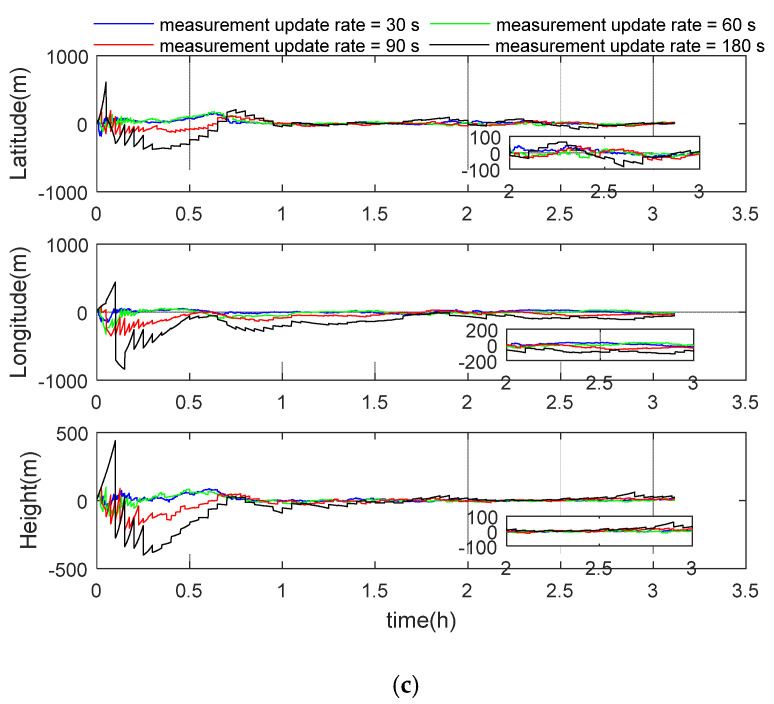
(**a**) Attitude error curve; (**b**) velocity error curve; (**c**) position error curve.

In order to evaluate the influence of different measurement update periods of gravity gradiometer on integrated navigation performance, four different measurement update periods (30, 60, 90, and 180 s) were selected for simulation experiments. Other parameters were set according to the benchmark parameters in Table 1. The simulation results are shown in the following Figure 4 and Table 3. According to Table 3, with the increase of measurement update period, the positioning error of the system gradually increases. The reason is that the observation information collected over the same time span gets less when using a larger measurement update period. When the measurement update period is less than 180 s, the position error in other directions is within 100 m, except for the longitude direction of 127 m. The 3D velocity errors for these four cases were 0.03 m/s, 0.05 m/s, 0.07 m/s, and 0.12 m/s, respectively, and the 3D position errors were 43 m, 52 m, 62 m, and 147 m.

### 5.3. Performance Analysis of Measurement Noise

**Figure 5:**
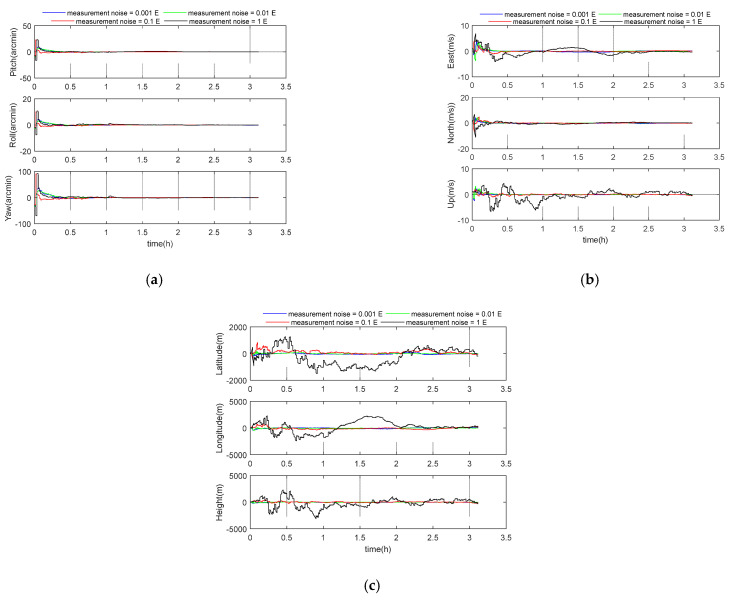
(**a**) Attitude error curve; (**b**) velocity error curve; (**c**) position error curve.

To investigate the effects of gradiometer noise on GGAN accuracy, different levels of gradiometer noise 1 mE, 0.01 E, 0.1 E, and 1 E were simulated. The 1 E and 0.1 E noise levels represent the precision of most current generation gradiometers, such as the ARKeX’s Exploration Gravity Gradiometer and the Gedex’s High-Definition Airborne Gravity Gradiometer. The 0.01 E noise level represents the precision of the latest gradiometers, such as the GOCE’s EGG. The 0.001 E noise level represents the precision of future grade gradiometers. Other parameters were set according to the benchmark parameters in Table 1. The simulation results are shown in the following Figure 5 and Table 4. According to Table 4, with the increase of measurement noise, the positioning performance of the system decreases rapidly. When the measurement noise is 1 E, the position error in longitude direction has exceeded 1000 m. The 3D velocity errors for these four cases were 0.05 m/s, 0.05 m/s, 0.21 m/s, and 1.90 m/s, respectively, and the 3D position errors were 40 m, 45 m, 227 m, and 1533 m.

### 5.4. Performance Analysis of Database Noise

**Figure 6:**
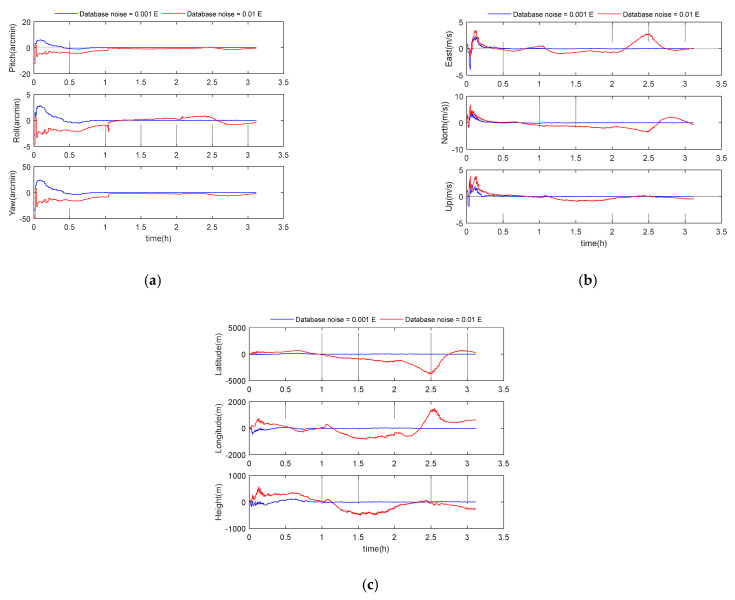
(**a**) Attitude error curve; (**b**) velocity error curve; (**c**) position error curve.

The accuracy of the established gravity gradient database is usually higher than that of the strapdown gradiometer. In order to reflect the impact of different database noise on the integrated navigation performance, two different database noises (0.001 and 0.01 E) were selected for our simulation experiment. Other parameters were set according to the benchmark parameters in Table 1. The simulation results are shown in the following Figure 6 and Table 5. According to Table 5, it can be seen that the gravity gradient database noise has a great impact on the performance of the GGAN system. When assuming a gradient noise of 0.001 E, the positioning performance is mostly better by one order of magnitude, compared to a noise level of 0.01 E. The 3D velocity errors for these two cases were 0.05 m/s and 1.86 m/s, respectively, and the 3D position errors were 60 m and 1509 m.

### 5.5. Performance Analysis of Initial Errors

**Figure 7:**
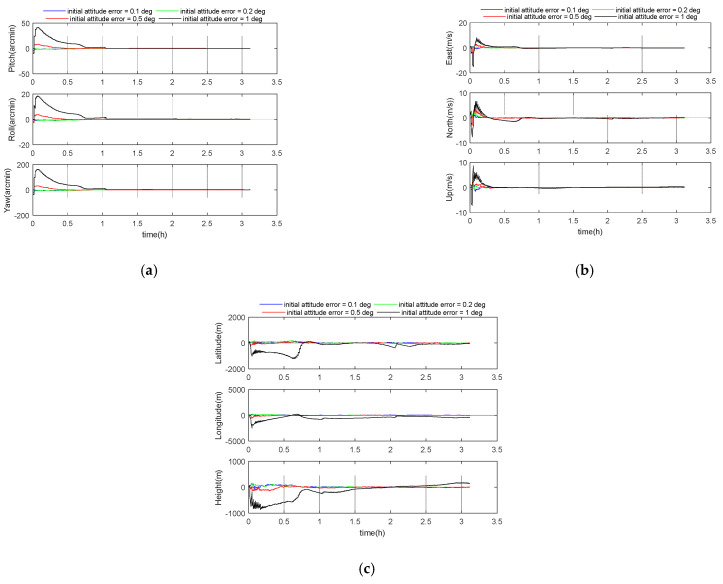
(**a**) Attitude error curve; (**b**) velocity error curve; (**c**) position error curve.

In order to reflect the influence of different initial SINS errors on the GGAN performance, the error parameters were divided into attitude error, velocity error, and position error. The initial attitude error was selected as 0.1, 0.2, 0.5, and 1 degree. Other parameters were set according to the benchmark parameters in Table 1. The simulation results are shown in the following Figure 7 and Table 6. It can be seen that the simulation converges in about an hour. After about 3 h of simulation, the 3D velocity errors for these four cases were 0.06 m/s, 0.06 m/s, 0.06 m/s, and 0.6 m/s, respectively, and the 3D position errors were 51 m, 51 m, 61 m, and 577 m. Therefore, when the initial attitude error is less than 0.5 deg, the influence of attitude error is not obvious.

**Figure 8:**
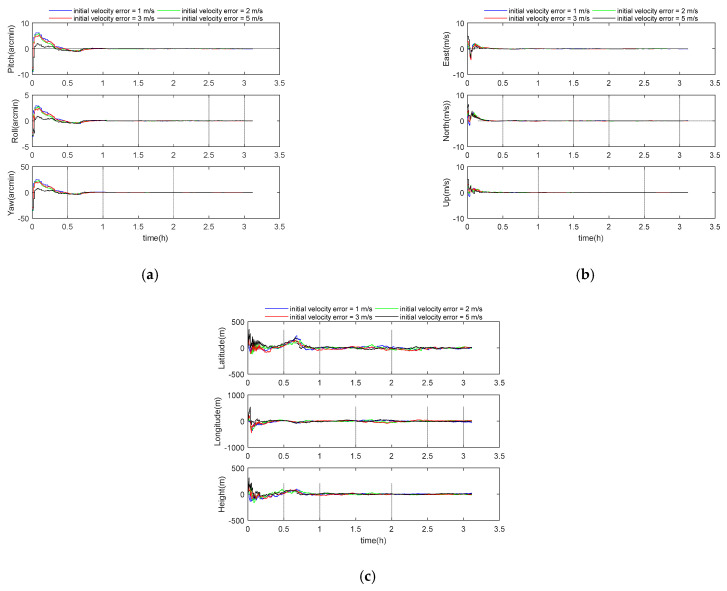
(**a**) Attitude error curve; (**b**) velocity error curve; (**c**) position error curve.

The initial velocity error was selected as 1, 2, 3, and 5 m/s. Other parameters were set according to the benchmark parameters in Table 1. The simulation results are shown in the following Figure 8 and Table 7. The 3D velocity errors for these four cases were 0.05 m/s, 0.05 m/s, 0.06 m/s, and 0.05 m/s, respectively, and the 3D position errors were 54 m, 45 m, 52 m, and 49 m. Therefore, under the given four cases of speed errors, the influence of speed error on the system is not obvious.

**Figure 9:**
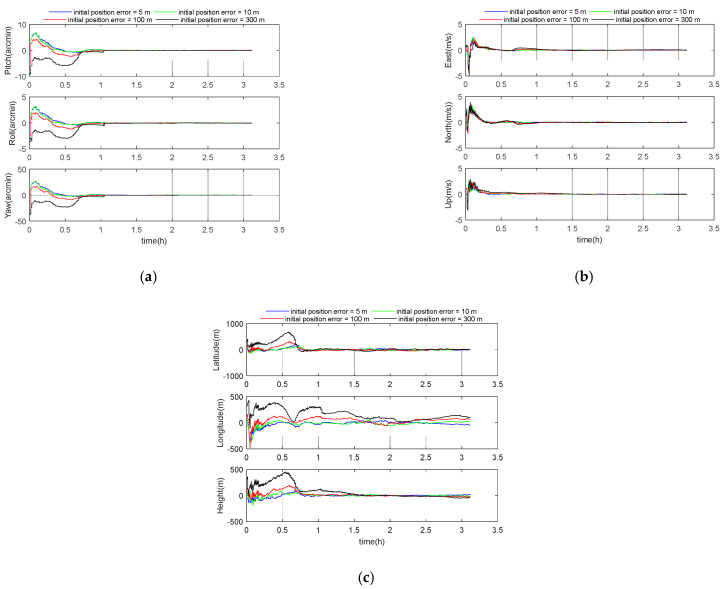
(**a**) Attitude error curve; (**b**) velocity error curve; (**c**) position error curve.

The initial position error was selected as 5, 10, 100, and 300 m. Other parameters were set according to the benchmark parameters in Table 1. The simulation results are shown in the following Figure 9 and Table 8. The 3D velocity errors for these four cases were 0.05 m/s, 0.05 m/s, 0.10 m/s, and 0.22 m/s, respectively, and the 3D position errors were 53 m, 44 m, 103 m, and 238 m.

According to Table 6, Table 7 and Table 8, we realize that the GGAN system is sensitive to the initial attitude error and initial position error of the inertial navigation system. On the other hand, the initial velocity error has little effect on the positioning performance of the system.

### 5.6. Performance Analysis of Inertial Navigation Level

**Figure 10:**
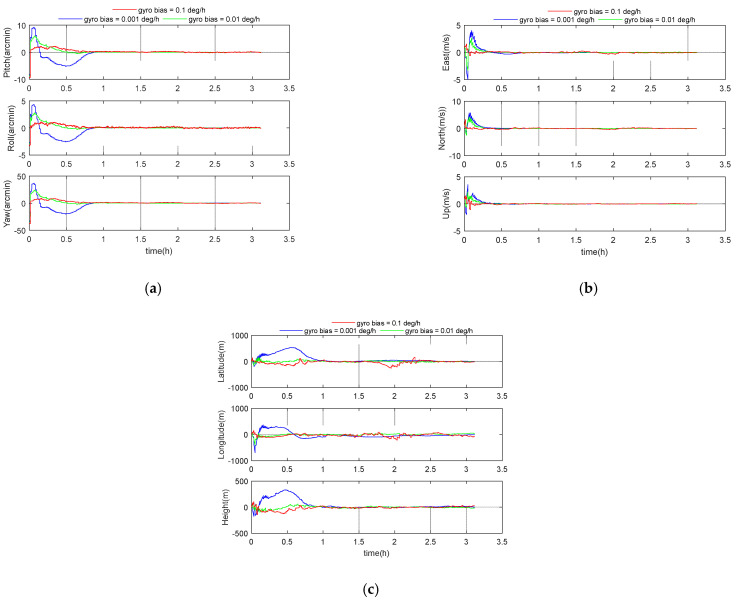
(**a**) Attitude error curve; (**b**) velocity error curve; (**c**) position error curve.

In order to reflect the influence of different inertial navigation levels on the integrated navigation performance, the gyro bias parameters were set at 1, 0.1, 0.01, and 0.001 degree per hour, respectively. Other parameters were set according to the benchmark parameters in Table 1. The simulation results are shown in the following Figure 10 and Table 9. It can be seen from Table 9, even if an inertial navigation system with a gyro bias of 0.1 degree per hour is adopted, the position error of the GGAN system is still controlled within 100 m. The 3D velocity errors for these three cases were 0.04 m/s, 0.05 m/s, and 0.17 m/s, respectively, and the 3D position errors were 28 m, 34 m, and 89 m. Therefore, the GGAN system can effectively reduce the requirements of inertial navigation device level under the condition of satisfying certain accuracy.

## 6. Conclusions

With the development of antisubmarine and submarine detection technology, in view of the role of submarines in the future information warfare, working out how to realize the submarine’s passive, autonomous, long-voyage, and high-precision navigation and positioning has become an urgent problem. The GGAN system makes it possible to realize high-precision positioning of a submarine over a long voyage time. The integrated navigation method of inertial/strapdown gravity gradiometer based on quaternion unscented Kalman filter is proposed in this paper, which can effectively restrain the divergence of inertial navigation error. The experimental results show that if the GGAN system is set according to the benchmark parameters, and after about 10 h of simulation the attitude error is less than 1 arcmin and the velocity error is less than 0.1 m/s, the position error is controlled within 33 m. With the increase of the measurement update period, the positioning error of the system gradually increases. When the measurement update period is less than 180 s, the position error in other directions is within 100 m, except for the longitude direction of 127.42 m. When the measurement noise is 1 E, the position error of longitude direction is more than 1000 m. The database noise has a great impact on the performance of the GGAN system. The noise of 0.001 E database is better than that of 0.01 E database, and the positioning performance of the system is mostly better than one order of magnitude. The GGAN system is sensitive to the initial attitude error and initial position error of the inertial navigation system, but the initial velocity error has little effect on the positioning performance of the system. Using GGAN, the system can reduce the requirements for the quality of the used inertial sensor. Therefore, the integrated navigation system based on gravity gradient can provide reliable and high-precision initial navigation parameters for other weapon systems, and provide a strong guarantee for the effectiveness of the entire combat platform.

## Figures and Tables

**Table 1:** Benchmark parameter settings.

Benchmark Parameter	Measurement Update Period	Measurement Noise	Database Noise	Initial Error			Gyro Bias
				Attitude	Velocity	Position	
Value	60 s	0.01 E	0.001 E	$0.5^{\circ }$	1 m/s	10 m	$0.01^{\circ }/h$

**Table 2:** Comparison of long voyage performance.

Mode	Attitude Errors (arcmin)			Velocity Error (m/s)			Position Error (m)		
	Pitch	Roll	Yaw	East	North	Up	Latitude	Longitude	Height
Pure inertial calculation	0.81	0.71	3.03	1.69	1.76	/	2640	2258	/
GGAN	0.28	0.18	0.72	0.06	0.09	0.03	33	29	17

**Table 3:** Simulation results under different measurement update periods.

Measurement Update Periods (s)	Attitude Errors (arcmin)			Velocity Error (m/s)				Position Error (m)			
	Pitch	Roll	Yaw	East	North	Up	3D	Latitude	Longitude	Height	3D
30	0.24	0.12	0.89	0.02	0.03	0.01	0.03	34	18	18	43
60	0.24	0.12	0.89	0.03	0.04	0.02	0.05	40	29	15	52
90	0.35	0.18	1.61	0.04	0.05	0.03	0.07	34	49	15	62
180	0.55	0.27	2.26	0.09	0.07	0.05	0.12	65	127	36	147

**Table 4:** Simulation results under different measurement noise.

Measurement Noise (E)	Attitude Errors (arcmin)			Velocity Error (m/s)				Position Error (m)			
	Pitch	Roll	Yaw	East	North	Up	3D	Latitude	Longitude	Height	3D
0.001	0.08	0.05	0.35	0.03	0.03	0.02	0.05	22	29	13	40
0.01	0.09	0.05	0.37	0.03	0.04	0.02	0.05	25	34	14	45
0.1	0.31	0.14	1.07	0.12	0.10	0.14	0.21	126	177	66	227
1	0.55	0.30	1.34	0.89	0.48	1.61	1.90	718	1087	807	1533

**Table 5:** Simulation results under different database noise.

Database Noise (E)	Attitude Errors (arcmin)			Velocity Error (m/s)				Position Error (m)			
	Pitch	Roll	Yaw	East	North	Up	3D	Latitude	Longitude	Height	3D
0.001	0.24	0.12	0.89	0.03	0.04	0.03	0.05	47	26	27	60
0.01	1.63	0.83	6.25	0.89	1.58	0.43	1.86	1379	559	249	1509

**Table 6:** Simulation results of different initial attitude errors.

Initial Attitude Errors (deg)	Attitude Errors (arcmin)			Velocity Error (m/s)				Position Error (m)			
	Pitch	Roll	Yaw	East	North	Up	3D	Latitude	Longitude	Height	3D
0.1	0.24	0.12	0.93	0.03	0.05	0.02	0.06	39	24	20	51
0.2	0.30	0.14	1.12	0.04	0.04	0.02	0.06	44	19	18	51
0.5	0.12	0.06	0.42	0.02	0.05	0.02	0.06	32	36	12	51
1	2.38	1.17	9.29	0.31	0.39	0.18	0.6	314	450	178	577

**Table 7:** Simulation results under different initial velocity errors.

Initial Velocity Errors (m/s)	Attitude Errors (arcmin)			Velocity Error (m/s)				Position Error (m)			
	Pitch	Roll	Yaw	East	North	Up	3D	Latitude	Longitude	Height	3D
1	0.22	0.11	0.80	0.03	0.04	0.02	0.05	45	22	20	54
2	0.19	0.09	0.71	0.03	0.04	0.02	0.05	35	23	16	45
3	0.23	0.11	0.83	0.03	0.05	0.02	0.06	39	27	19	52
5	0.24	0.12	0.89	0.03	0.03	0.02	0.05	38	24	18	49

**Table 8:** Simulation results under different initial position errors.

Initial Position Errors (m)	Attitude Errors (arcmin)			Velocity Error (m/s)				Position Error (m)			
	Pitch	Roll	Yaw	East	North	Up	3D	Latitude	Longitude	Height	3D
5	0.20	0.10	0.73	0.03	0.04	0.02	0.05	43	23	19	53
10	0.17	0.08	0.62	0.03	0.04	0.02	0.05	33	23	15	44
100	0.54	0.27	2.08	0.06	0.07	0.04	0.10	70	60	44	103
300	1.25	0.63	4.89	0.14	0.11	0.13	0.22	151	150	106	238

**Table 9:** Simulation results of different inertial navigation levels.

Gyro Bias $\left({}^{\circ }/h\right)$	Attitude Errors (arcmin)			Velocity Error (m/s)				Position Error (m)			
	Pitch	Roll	Yaw	East	North	Up	3D	Latitude	Longitude	Height	3D
0.001	0.08	0.05	0.27	0.03	0.02	0.02	0.04	19	15	13	28
0.01	0.09	0.05	0.33	0.03	0.04	0.02	0.05	23	20	14	34
0.1	0.25	0.14	0.97	0.10	0.14	0.03	0.17	69	54	16	89

## Data Availability

Data sharing not applicable. No new data were created or analyzed in this study. Data sharing is not applicable to this article.

## Appendix A

The normalized gravitational potential function V(r,θ,λ) can be expressed in the form of higher-order spherical harmonic function as follows:

(A1) $$V(r,\theta ,\lambda )=\frac{GM}{a}\sum_{n=0}^{N}\sum_{m=0}^{n}{\left(\frac{a}{r}\right)}^{n+1}{\bar{P}}_{n,m}(\cos\theta )[{\bar{C}}_{n,m}\cos(m\lambda )+{\bar{S}}_{n,m}\sin(m\lambda )]$$

Here, GM is the gravitational constant multiplied by the Earth’s mass, where θ=π2−ψ, and r, ψ, λ represent the geocentric distance, the latitude, and longitude, and a is the radius of the Earth. N is the maximum value of the degree n and order m. ${\bar{P}}_{n}^{m}(\cos\theta )$ is the normalized Legendre associated function of the degree n and order m. ${\bar{C}}_{n,m}$ and ${\bar{S}}_{n,m}$ are the harmonic geopotential coefficients with degree n and order m.

According to [27], the calculation formula of gravity gradients reads as follows:

(A2) $$V_{x_{i}x_{j}}=GM\frac{\partial^{2}\frac{1}{r}}{\partial x_{i}x_{j}}+\frac{GM}{a^{2}}\sum_{n=2}^{N+1}\sum_{m=0}^{n}{\left(\frac{a}{r}\right)}^{n+1}{\bar{P}}_{n,m}(\cos\theta )[{\bar{C}}_{n,m}^{x_{i}x_{j}}\cos(m\lambda )+{\bar{S}}_{n,m}^{x_{i}x_{j}}\sin(m\lambda )]$$

where ${\bar{C}}_{n,m}^{x_{i}x_{i}}$ and ${\bar{S}}_{n,m}^{x_{i}x_{i}}$ can be obtained from [28]. The specific calculation formula of $\partial^{2}\frac{1}{r}/\partial x_{i}x_{j}$ is as follows:

(A3) $$\frac{\partial^{2}\frac{1}{r}}{\partial x_{i}x_{j}}=\left\{\begin{array}{ll} \frac{3x_{i}{}^{2}-r^{2}}{r^{5}} & \mathrm{if}\;i=j\\ \frac{3x_{i}x_{j}}{r^{5}} & \mathrm{if}\;i\neq j \end{array}\right.$$

The transformation of the gravity gradient tensor matrix between the body coordinate system and the navigation coordinate system is as follows:

(A4) $$V_{b}=C_{n}^{b}V_{n}{\left(C_{n}^{b}\right)}^{T}=C_{n}^{b}V_{n}C_{b}^{n}$$
